# A suboptimal maternal diet combined with accelerated postnatal growth results in an altered aging profile in the thymus of male rats

**DOI:** 10.1096/fj.201701350RR

**Published:** 2018-07-05

**Authors:** Jane L. Tarry-Adkins, Catherine E. Aiken, Thomas J. Ashmore, Denise S. Fernandez-Twinn, Jian-Hua Chen, Susan E. Ozanne

**Affiliations:** University of Cambridge Metabolic Research Laboratories and Medical Research Council (MRC) Metabolic Diseases Unit, Wellcome Trust–MRC Institute of Metabolic Science, Addenbrooke’s Treatment Centre, Addenbrooke’s Hospital, Cambridge, United Kingdom

**Keywords:** developmental programming, involution, immunosenescence

## Abstract

Reduced fetal nutrition and rapid postnatal growth accelerates the aging phenotype in many organ systems; however, effects on the immune system are unclear. We addressed this by studying the thymus from a rat model of developmental programming. The recuperated group was generated by *in utero* protein restriction, followed by cross-fostering to control-fed mothers, and were then compared with controls. Fat infiltration and adipocyte size increased with age (*P* < 0.001) and in recuperated thymi (*P* < 0.05). Cortex/medulla ratio decreased with age (*P* < 0.001) and decreased (*P* < 0.05) in 12-mo recuperated thymi. Age-associated decreases in thymic–epithelial cell (*P* < 0.01) and thymocyte markers (*P* < 0.01) were observed in both groups and was decreased (*P* < 0.05) in recuperated thymi. These data demonstrate effects of developmental programming upon thymic involution. The recuperated group had longer thymic telomeres than controls (*P* < 0.001) at 22 d and at 3 mo, which was associated with increased expression of telomere-length maintenance molecules [telomerase RNA component (*Terc*; *P* < 0.01), *P23* (*P* = 0.02), and *Ku70* and *Ku80* (*P* < 0.01)]. By 12 mo, recuperated offspring had shorter thymic telomeres than controls had (*P* < 0.001) and reduced DNA damage-response markers [(*DNA-PKcs*, *Mre11* (*P* < 0.01), *Xrcc4* (*P* = 0.02), and *γ-H2ax* (*P* < 0.001], suggesting failure of earlier compensatory responses. Our results suggest that low birth weight with rapid postnatal growth results in premature thymic maturation, resulting in accelerated thymic aging. This could lead to increased age-associated vulnerability to infection.—Tarry-Adkins, J. L., Aiken, C. E., Ashmore, T. J., Fernandez-Twinn, D. S., Chen, J.-H., Ozanne, S. E. A suboptimal maternal diet combined with accelerated postnatal growth results in an altered aging profile in the thymus of male rats.

Immune insufficiency and the rate of infections are known to increase considerably with age (1). With the number of people 65 yr or older predicted to increase from 524 million in 2010 to 1.5 billion in 2050 (2), we are likely to see a huge impact on the incidence of immune insufficiency and associated diseases among the elderly population. Immune insufficiency has been overtly associated with the top 5 causes of death in a population older than 65 yr (3). Therefore, there is a real need to understand potential underlying mechanisms that could contribute to age-associated immune dysfunction.

The thrifty phenotype hypothesis was postulated >25 yr ago by Hales and Barker (4) to explain the phenomenon of increased risk of developing age-associated diseases, such as cardiovascular disease and type-2 diabetes, when individuals were exposed to conditions of suboptimal maternal nutrition during fetal life. Under those conditions, the fetus is developmentally programmed,”resulting in structural changes to organ development and adaptations to its metabolism to ensure immediate postnatal survival of the organism. This can occur through the sparing of certain vital organs, especially the brain, at the expense of other organs, including the heart, pancreas, kidney, and skeletal muscle, resulting in compromised structures and functions of those organs. Furthermore, a suboptimal maternal environment combined with a mismatched postnatal milieu can further exacerbate the increased risk of the development of a wide range of age-associated diseases in the offspring later in life (2). Despite decline in immune function having an important role in the development of many age-associated diseases, it has been poorly studied in the context of developmental programming.

The thymus is a primary lymphoid organ that has a fundamental role in the development of self-restricted, immunocompetent T cells (5). Thymic development occurs during the late fetal/early postnatal stages of mammalian growth (6) and is, therefore, acutely sensitive to changes in the early life environment (7). Prenatal undernutrition resulting in low birth weight has been associated with a decline in thymic and immune function in Filipino adolescents (8, 9), and that has been recapitulated in a cohort of low-birth weight Bangladeshi children (10, 11) as well as a cohort of small-for-gestational age and intrauterine growth-restricted individuals (12). Thus, a suboptimal *in utero* environment influences thymic development, and that, in turn, may be mediated by maternal nutritional status.

During the aging process, the thymus undergoes involution, which is one of the most dramatic changes seen in the aging immune system. During this process, changes in thymic ultrastructure, including thinning of the thymic cortex and disorganization of the cortical and medullary regions of the thymus occur. In addition, thymic epithelial cell (TEC) numbers decline and are replaced with adipocytes, which infiltrate the aging thymus. Eventually, adipocytes constitute the bulk of the thymic cellular space (13). Markers of TECs include transcription factor 3 (*Tcf3*), keratin 8 (*Krt8*), *Il7*, and forkhead box N1 (*Foxn1*) and are essential for maintenance of thymic architecture, development, function, maintenance, and regeneration (14). The eventual outcome of thymic involution is a reduction in naive T-cell output, resulting in immunosenescence, which puts an individual at greater risk for developing an infectious disease and increased risk of morbidity and mortality from infectious diseases (15, 16). We have previously reported that early life nutrition and, consequently, growth trajectories influence thymic growth; specifically, a normal birth weight followed by slow growth during lactation is associated with increased thymic growth and enhanced mitotic activity (17).

Telomeres are the physical ends of linear chromosomes, the length of which is a robust marker of cellular aging and senescence (18, 19). They have been shown to be involved in longevity regulation in a range of species (20). In mammals, telomeres are composed of a variable number of tandem repeats of DNA of the form (TTAGGG)n. In normal human somatic cells, because of inherent limitations in the mechanics of DNA replication, telomeres shorten with each cell division (in the absence of telomerase). When telomeres reach a critically short length, the telomere becomes dysfunctional and is characterized as a double-stranded DNA (dsDNA) break. These dsDNA breaks induce a series of DNA damage checkpoint proteins, including P53, P21, and P16^INK^, as part of the cellular senescence pathway (21). Reactive oxygen species (ROS) are known to damage DNA, proteins, and lipids if the cellular antioxidant defense capacity is insufficient to mop up excess ROS. Telomeric DNA is particularly susceptible to ROS damage because of its guanine-rich sequence (TTAGGG) (22). Major pathways of ROS generation, include the uncoupling of the mitochondrial electron transport chain, increased xanthine oxidase activity, and activation of NADPH oxidase enzymes.

This study aimed to investigate the effects of maternal protein restriction during pregnancy, followed by accelerated postnatal growth (recuperated phenotype), on measures of thymic aging in male rats. The outcome measures included histologic assessment of thymic involution, expression of TECs and thymocytes, telomere length, expression of telomere-length maintenance proteins, markers of DNA damage, markers of oxidative stress, antioxidant-defense capacity, cellular senescence, and markers of mitochondrial dysfunction.

## MATERIALS AND METHODS

This research was conducted under the Animals (Scientific Procedures) Act 1986 Amendment Regulations of 2012, following ethical review by the University of Cambridge Animal Welfare and Ethical Review Board. Stock animals were purchased from Charles River Laboratories (Wilmington, MA, USA). Dams were produced from in-house breeding of stock animals. Pregnant Wistar rats (R*attus norvegicus*) were maintained at 22°C, on a controlled 12:12-h light/dark cycle, in specific pathogen-free housing in individually ventilated cages with environmental enrichment. The dams were maintained on a 20% protein diet (control) or an isocaloric low-protein (8%) diet, as previously described by Snoeck *et al.* (23). Access to diets and water was provided *ad libitum*. Diets were purchased from Arie Blok (Woerden, The Netherlands).

The day of birth was recorded as d 1 of postnatal life. Pups born to low-protein diet-fed dams were cross-fostered to control-fed mothers on postnatal d 3 to create a recuperated litter. Each recuperated litter was standardized to 4 male pups at random to maximize their plane of nutrition. The control group was the offspring of mothers fed the standard 20% protein diet and suckled by 20% protein-fed dams. Each control litter was culled to 8 male pups as a standard. To minimize stress during crossfostering, pups were transferred with some of their own bedding. Body weights were recorded on postnatal d 3, 7, 10, 14, and 21 and at 3 and 12 mo of age. Time points until 21 d reflect the average male pup weight in the litter. One male offspring/litter was culled at 22 d old, another at 3 mo old, and another male was culled at 12 mo old. All animals were killed by CO_2_ asphyxiation at ∼10:00 am after withholding food overnight. At postmortem, the whole thymus was removed, weighed, and snap frozen in liquid nitrogen, and then, stored at −80°C until analysis.

### Histologic assessment

Frozen thymus tissue was fixed in formalin and then processed to paraffin and embedded and sectioned at 5 µm on a Microtome (RM2235; Leica Camera, Wetzlar, Germany). Sections were stained with hematoxylin and eosin for general histologic assessment and with Picro Sirius Red for assessment of fibrosis. Sudan B Black staining was used as a proxy marker for senescent cells [the methodology was the same as that used in Georgakopoulou *et al.* (24)]. The images were taken at ×20 magnification on an Axioscan.Z1 slide scanner (Carl Zeiss, Oberkochen, Germany), using Zen software (Carl Zeiss). Analysis of sections was conducted with the Halo image software package (Indica Labs, Corrales, NM, USA). Gross thymus ultrastructure analysis (areas of whole thymus, cortex, medulla, and adipose tissue) was performed with specific-area annotations. Classification analysis (in which the software was programmed to recognize fibrotic tissues *vs.* other thymic tissue) was established to calculate the degree of thymic fibrosis. A further classifier was set up to detect Sudan Black B–positive cells *vs.* other thymus tissue to determine cell senescence area. Resolution in all cases was set at 2 µm/pixel. To measure adipocyte size and number, a classifier algorithm with the vacuole/steatosis module was used. The following parameters were set: vacuole diameter (minimum: 2000; maximum 200,000), vacuole contrast (50), vacuole-intensity threshold (0.808), minimum vacuole roundness (0.272), minimum edge fragment area (1000), vacuole-segment aggressiveness (0.75), and maximum-segmentation length (15).

### Telomere-length measurement

High-MW DNA was extracted with phenol/chloroform DNA methodology. DNA quantity and purity were determined with a spectrophotometer (Nanodrop Technologies, Wilmington, DE, USA) (25). DNA integrity was confirmed by agarose gel electrophoresis. DNA (1.2 µg) was digested by *Hin*FI and *Rsa*I restriction enzymes at 37°C for 2 h, separated by pulsed-field gel electrophoresis and transferred to nylon membranes by Southern blotting (25). Standard undigested and digested genomic DNA from a 22-d-old control animal was also included on each gel to verify digestion efficiency (25). Telomere length was measured with Telo TAGGG telomere-length assays (Roche Diagnostics, Basel, Switzerland). Telomere signals were analyzed with Photoshop software (Adobe Systems, San Jose, CA, USA) and Alpha Ease Software (Alpha Innotech, San Leandro, CA, USA). Telomere length was quantified where the percentage of intensity (percentage of telomere length) of the telomeric signal was determined in 4 molecular-size regions, as defined by MW markers (25, 26).

### Gene expression

RNA was extracted using an RNeasy Plus Mini Kit (Qiagen, Hilden, Germany) following the manufacturer’s instructions. A DNase digestion step was performed to remove genomic DNA contamination. RNA (1 µg) was used to synthesize cDNA using oligo-dT primers and Moloney murine leukemia virus reverse transcriptase (Promega, Madison, WI, USA). Gene expression was determined with custom-designed primers (MilliporeSigma, Burlington, MA, USA) and SYBR Green reagents (Thermo Fisher Scientific, Waltham, MA, USA). Primer sequences are presented in Supplemental Table 1. Quantification of gene expression was performed on a Step One Plus RT-PCR machine (Thermo Fisher Scientific). Equal efficiency of the reverse transcription of RNA from all groups was confirmed through quantification of expression from the housekeeping gene *Ppia.* Expression of *Ppia* did not differ between groups [means ± sem, 22-d control (53,154 ± 5671), 22-d recuperated (54,504 ± 7419); 3-mo control (48,379 ± 9299), 3-mo recuperated (52,450 ± 9252); 12-mo control (46,596 ± 6901), 12-mo recuperated (39,935 ± 3340) copy numbers; effect of maternal diet: *P* = 0.9; effect of age: *P* = 0.32; interaction of maternal diet and age: *P* = 0.7].

### Statistical analysis

Statistical analyses were performed with R software (v.3.1.0; R Foundation for Statistical Computing, Vienna, Austria), unless otherwise stated. A series of linear regression models were used to determine the effect of maternal diet, age, and any interaction between maternal diet and age on RNA copy number on a gene-by-gene basis. To correct for multiple-hypothesis testing, significancevalues were transformed to take account of the false-discovery rates using the *p.adjust* function in the R statistical package. Linear-regression models that included effects of maternal diet and age were used to analyze telomere-length data. The statistical significance and the total variation within the data attributable to each covariate are reported. Raw gene-expression values for genetic markers of thymocyte and TEC lineage were transformed to *z* scores to allow direct comparison of expression levels between genes. Hierarchical linear models were constructed, with fixed effects for age and maternal diet, an interaction term between age and maternal diet, and a random effect for genes. Models were run for markers of thymocyte lineage and TEC lineage separately and were compared with null models to obtain the *P* values for the statistical significance of each tested effect. The ratio of *z* scores for genetic markers of thymocyte lineage and TEC lineage was then calculated. A linear-regression model, with independent effects of age and maternal diet and an interaction term between age and maternal diet, was specified. All data were checked for normal distribution. In all cases, *n* refers to the number of litters (with 1 animal used from each litter at each time point). Because the linear-regression analysis for gene expression for all genes revealed no significant difference between 22 d and 3 mo of age (adjusted *P* values between 0.3 and 0.99) (Supplemental Table 1), the gene-expression data are reported as a comparison only between 3 and 12 mo of age. Anthropomorphic measurements and histologic assessments were analyzed by 2-way ANOVA with maternal diet and offspring age as the independent variables and Duncan’s *post hoc* testing, where appropriate (Statistica; Tibco Software, Palo Alto, CA, USA). Data are represented as means ± sem. Where *P* values are reported, an α level <0.05 was considered statistically significant.

## RESULTS

### Effect of maternal diet and age upon anthropometric measurements

At postnatal d 3, the recuperated pups were lighter than the control pups were (6.3 ± 0.2 compared with 7.4 ± 0.2 g; *P* < 0.001) because their biologic mothers were fed a low-protein diet during pregnancy. However, they underwent rapid postnatal catch-up growth because of the increased plane of nutrition from the control-fed foster mothers during lactation, such that by postnatal d 22, there was no difference in body weight between the groups (control 49 ± 1.3 g *vs.* recuperated 51 ± 1.7 g). That similarity was maintained at 3 and 12 mo of age. An age-associated increase (*P* < 0.001) in weight was observed in both groups (**Table 1**).

**TABLE 1:** The effect of maternal nutrition and accelerated postnatal growth and aging upon body weight and thymic weight of male rats

Maternal diet	Age (mo)	Body weight (g)	Thymic weight (g)	Cortical area (µm^2^)	Medullary area (µm^2^)
Control	3	482 ± 12	0.73 ± 0.03	98,759,092 ± 8,634,857	31,337,794 ± 2,807,553
Recuperated	3	476 ± 8	0.69 ± 0.02	99,985,148 ± 13,071,532	26,885,423 ± 2,565,486
Control	12	956 ± 25***	1.3 ± 0.06***	28,534,871 ± 5,603,828***	9,531,644 ± 2,028,512***
Recuperated	12	920 ± 29***	1.3 ± 0.07***	35,984,906 ± 4,795,040***	18,814,493 ± 2,363,878***

*** *P* < 0.001 (control 3 mo old *vs.* control 12 mo old; recuperated 3 mo old *vs.* recuperated 12 mo old). Data are represented as means ± sem.

### Effect of maternal diet and age upon thymic weight and ultrastructure

**Figure 1:**
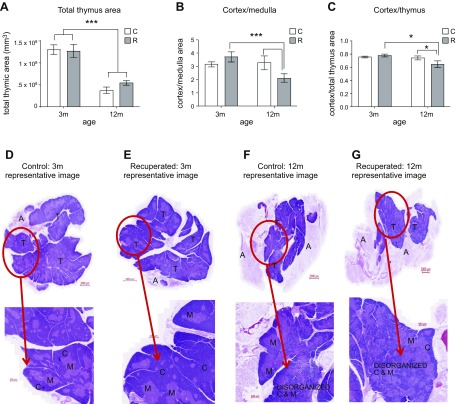
The effect of *in utero* protein restriction, accelerated postnatal growth, and aging upon total thymus area (*A*), thymic cortex to medulla area ratio (*B*), cortex to total thymus area ratio (*C*), 3-mo control (*D*) (representative image), 3-mo recuperated (*E*) (representative image), 12-mo control (*F*) (representative image), and 12-mo recuperated (*G*) (representative image). Results are expressed as means ± sem; *n* = 8/group. C, control; R, recuperated. *P < 0.05, ***P < 0.001.

**Figure 2:**
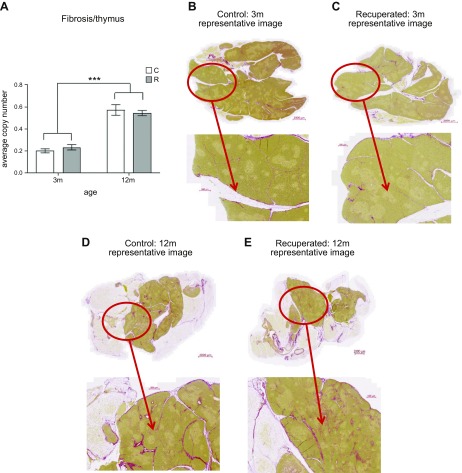
The effect of *in utero* protein restriction, accelerated postnatal growth, and aging upon fibrosis in the thymus of 3- and 12-mo-old, male rats. Fibrosis/thymus area (*A*), 3-mo control (*B*) (representative image), 3-mo recuperated (*C*) (representative image), 12-mo control (*D*) (representative image), and 12-mo recuperated (*E*) (representative image) results are shown. Results are expressed as means ± sem; *n* = 8/group. C, control; R, recuperated . ***P < 0.001.

With age, absolute thymic mass increased (*P* < 0.001; Table 1), and total thymic area (**Fig. 1*A***), cortical area, and medullary area all decreased (*P* < 0.001; Table 1). However, there was no effect of maternal diet upon thymic weight (Table 1) or upon cortical area (Table 1). However, there was an interaction between maternal diet and age (*P* < 0.05) in relation to medullary area, with the age-associated decrease in medullary area less pronounced in recuperated thymi compared with controls (Table 1). The ratio of thymic cortex to medulla area (C/M) was calculated as a marker of involution. An overall reduction (*P* < 0.01) in that ratio was observed with age. An interaction (*P* < 0.05) between maternal diet and age highlighted that the C/M in recuperated thymi decreased more with age than it did in control thymi (Fig. 1*B*). Those data were highlighted by the lower (*P* < 0.05) C/M ratio in recuperated thymi compared with control thymi at 12 mo of age (Fig. 1*B*). A ratio of cortex to total thymus area (C/T) was also calculated as an index of cortical thinning in the thymus. An age-associated decrease in C/T was observed. An interaction (*P* < 0.05) between maternal diet and age revealed that the C/T ratio decreased more with age in recuperated thymi than it did in controls (Fig. 1*C*). The age-associated disorganization of the thymic cortex and medulla was apparent visually (Fig. 1*D*–*G* for representative images). As expected, there was also an age-associated increase in fibrosis (**Fig. 2*A***). There was no effect of maternal diet upon fibrosis (Fig. 2*B*–*E* for representative images).

### Effect of maternal diet and age upon indices of fat infiltration

**Figure 3:**
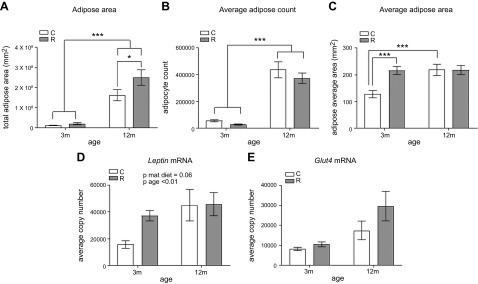
The effect of *in utero* protein restriction, accelerated postnatal growth, and aging upon the expression of markers of fat accumulation in the thymus of 3- and 12-mo-old, male rats. Total adipose area (*A*), average adipocyte count (*B*), average adipocyte area (*C*), and *Lep* (*D*) and *Glut4* (*E*) mRNA expression are shown. Results are expressed as means ± sem. C, control; R, recuperated; *n* = 8/group. For gene expression data, overall effect of maternal diet (*P* mat diet) <0.06, and overall effect of age (*P* age) <0.01. *P < 0.05, ***P < 0.001 for histologic data.

With age, adipose tissue area (*P* < 0.001; **Fig. 3*A***), adipocyte number (*P* < 0.001; Fig. 3*B*), and average adipocyte area (*P* < 0.01) increased (Fig. 3*C*). Maternal diet resulted in increased (*P* < 0.001) adipose tissue area (Fig. 3*A*); however, adipocyte number was unaffected by maternal diet (Fig. 3*B*). There was an overall effect of maternal diet upon adipocyte area (*P* < 0.05), with larger adipocytes observed in recuperated thymi compared with controls (Fig. 3*C*). Larger adipocytes (*P* < 0.01) were also observed with age (Fig. 3*C*), which was more pronounced in control thymi than it was in recuperated thymi (Fig. 3*C*), as illustrated by the interaction between maternal diet and age (*P* < 0.05). Representative images of those data can be found in Fig. 1*D*–*G*. Thymic leptin (*Lep*) and glucose transporter 4 (*Glut4*) expression were measured as molecular indicators of fat infiltration. There was a borderline overall effect (*P* = 0.06) of maternal diet upon *Lep* mRNA expression (Fig. 3*D*), with increased *Lep* in recuperated thymi compared with that of controls and an age-associated increase (*P* < 0.01) in *Lep* expression (Fig. 3*D*). *Glut4* expression was not significantly altered by maternal diet or age (Fig. 3*E*).

### Effect of maternal diet and age upon molecular markers of TECs and thymocytes

**Figure 4:**
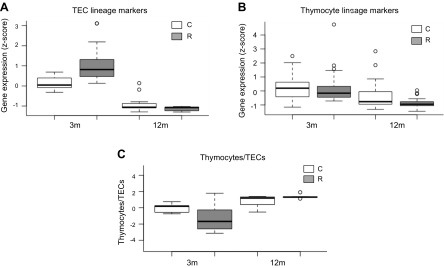
The effect of *in utero* protein restriction, accelerated postnatal growth, and aging upon the expression of TEC and thymocyte markers in 3- and 12 mo-old male rats. *A*) Geometric mean of markers of TEC lineage. *B*) Geometric mean of markers of thymocyte lineage. *C*) Ratio of TEC to thymocyte markers. Results are expressed as means ± sem; *n* = 8/group. C, control; R, recuperated. Interaction between maternal diet and age (*P* int) <0.05. Overall effect of maternal diet <0.01. Overall effect of age <0.01. TEC/thymocyte ratio is expressed as a geometric mean.

There was an overall effect of age (*P* < 0.01) in reduced expression of TEC markers *Tcf3*, *Krt8*, *Il7*, and *Foxn1* (**Table 2**). There was an interaction between maternal diet and age for *Tcf3* (*P* < 0.05), *Il7* (*P* = 0.02), and *Foxn1* (*P* < 0.01) expression, highlighting a greater age-associated decline in expression of those genes in recuperated thymi (Table 2). These findings were recapitulated when all genes were expressed as overall geometric means (effect of age, *P* = 0.01; interaction between maternal diet and age, *P* = 0.05; **Fig. 4*A***). Thymocyte markers *Cd8* (*P* < 0.05) and *Cd69* (*P* = 0.1) declined with age and were reduced (*Cd8*, *P* < 0.05; *Cd69*, *P* = 0.06) in recuperated thymi compared with controls (Table 2). No effect of age or maternal diet was observed upon *Cd44* expression (Table 2). When expressed as geometric means, markers of thymocytes were reduced (*P* < 0.01) with age (Fig. 4*B*) and were also decreased (*P* < 0.05) in recuperated thymi compared with controls (Fig. 4*B*). There was a significant (*P* < 0.01) effect of age upon the ratio of expression of TEC to thymocyte lineage markers, with age increasing that ratio. There was no overall independent effect of maternal diet on the ratio of expression of TEC to thymocyte lineage markers (Fig. 4*C*).

**TABLE 2:** The effect of maternal diet and accelerated postnatal growth upon individual markers of TEC and thymocyte lineage in thymi from male rats

Gene	Control 3 mo	Recuperated 3 mo	Control 12 mo	Recuperated 12 mo	Effect of age	Effect of maternal diet	Interaction effect
*Trf3*	12,887 ± 2301	17,429 ± 1887	10,356 ± 3770	2445 ± 570	*P* < 0.01	NS	*P* = 0.05
*Krt8*	13,885 ± 1721	15,988 ± 656	1524 ± 458	655 ± 97	*P* < 0.01	NS	NS
*Il7*	6211 ± 496	8978 ± 617	854 ± 371	106 ± 68	*P* < 0.01	NS	*P* < 0.05
*Foxn1*	2713 ± 260	5722 ± 260	73 ± 32	54 ± 12	*P* < 0.01	NS	*P* < 0.01
*Cd8*	93,320 ± 23,343	34,392 ± 4097	9089 ± 6281	303 ± 125	*P* < 0.01	*P* < 0.05	NS
*Cd44*	260,769 ± 82,995	315,584 ± 93,850	153,302 ± 62,999	72,360 ± 43,091	NS	NS	*P* = 0.10
*Cd69*	564,696 ± 150,429	220,410 ± 33,127	306,656 ± 124,432	8058 ± 3620	*P* = 0.10	*P* = 0.06	NS

Interaction between maternal diet and age: *Tcf3* (*P* = 0.05), *Il7* (*P* = 0.02), and *Foxn1* (*P* < 0.01), demonstrating a greater age-associated decline in expression of those genes in recuperated thymi. NS, not significant.

### Effect of maternal diet and age upon thymic telomere length

**Figure 5:**
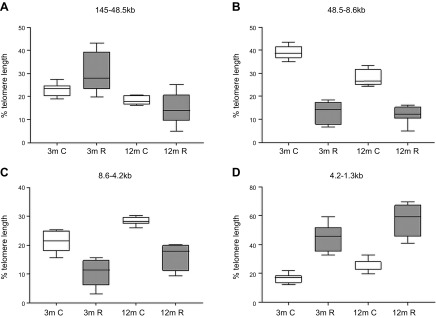
The effect of *in utero* protein restriction, accelerated postnatal growth, and aging upon the thymic telomere length in 22-d, 3-mo, and 12-mo male rats. 145-48.5kb (*A*), 48.5-8.6kb (*B*), 8.6-4.2kb (*C*), and 4.2-1.3kb (*D*). Results are expressed as median ± interquartile range and total range, excluding outliers. C, control; R, recuperated; *n* = 8/group.

Significant variation because of age was observed in very long (145–48.5 kb; *P* < 0.01), intermediate-long (48.5–8.6 kb; *P* < 0.001), intermediate-short (8.6–4.2 kb; *P* < 0.001), and very short (4.2–1.3 kb; *P* < 0.001) telomeres (**Fig. 5*A–D*** and **Table 3**). Specifically, more intermediate-long telomeres and fewer intermediate-short telomeres were observed between 22 d and 3 mo in the control group (suggesting increased telomere length in control thymi between those ages). Control telomere length then decreased between 3 and 12 mo with fewer intermediate-long telomeres and more intermediate-short telomeres (Fig. 5*B*, *C*). In the recuperated group, there was an age-associated decrease in telomere length, with fewer long (145–48.5 and 48.5–8.5 kb) telomeres in 12-mo animals compared with the results at 3 mo and at 22 d (Fig. 5*A*, *B*). More very short (4.2–1.3 kb) telomeres were also observed in old, compared with young, recuperated rats (Fig. 5*D*). Significant variation because of maternal diet was also observed in very long (145–48.5 kb; *P* < 0.001), intermediate-long (48.5–8.6 kb; *P* < 0.001), and very short (4.2–1.3 kb; *P* < 0.001) telomeres (Table 3). Specifically, the percentage of very long telomeres increased in recuperated thymi, compared with controls in early life (Fig. 5*A*). That effect was lost in later life. Frequency of intermediate-long (48.5–8.6 kb) and intermediate-short (8.6–4.2 kb) telomeres was less in recuperated thymi compared with controls (Fig. 5*B*, *C*). Frequency of very short telomeres (4.2–1.3 kb) was increased in recuperated thymi compared with controls at all ages (Fig. 5*D*).

**TABLE 3:** Variation due to age and maternal diet in each telomere length

Telomere length	Estimate of total variation from maternal diet (%)	Estimate of total variation from age (12 mo) (%)
Very long telomeres (14548.5 kb)	6.5**	11.7***
Long telomeres (48.5–8.6 kb)	17.1***	8.3***
Short telomeres (4.2–1.3 kb)	12.1***	0.7
Very short telomere (4.2–1.3 kb)	22.7***	19.3***

** *P* < 0.01, ***P < 0.001. Data represent means ± sem. Total telomere length for each sample was divided into 4 categories (145–48.5, 48.5–8.6, 4.2–1.3, and 4.2–1.3 kb), and the percentage of each sample’s telomeres falling into each category was calculated. Those percentages were then used in linear-regression models to determine the influence of maternal diet and age, respectively, on each of the 4 telomere-length groups. The variance attributable to each factor at each length is reported along with the strength of the association (*P*) from the linear-regression model.

### Effect of maternal diet and age upon markers of cellular senescence

There was no effect of maternal diet or age on thymic gene expression on either *P53* (3-mo controls: 21,693 ± 1564, 3-mo recuperated: 24,193 ± 1564, 12-mo controls: 23,875 ± 5240, and 12-mo recuperated: 16,952 ± 2522 copy number) or *P21* (3-mo controls: 3847 ± 483, 3-mo recuperated: 3382 ± 472, 12-mo controls: 3357 ± 759, and 12-mo recuperated: 2207 ± 438 copy number). Sudan Black B, a proxy histologic stain for senescent cells demonstrated an overall age-associated increase in senescent cells; however, no effect of maternal diet was observed (3-mo controls: not performed, 3-mo recuperated: not detectable, 12-mo controls: 1.365 ± 0.54 mm^2^, and 12-mo recuperated 1.797 ± 0.85 mm^2^) (*P* = 0.66; Student’s *t* test, 2-tail). (Representative images of these data can be found in Supplemental Fig. 1).

### Effect of maternal diet and age upon molecules associated with telomere length regulation

#### Telomerase complex molecules

**Figure 6:**
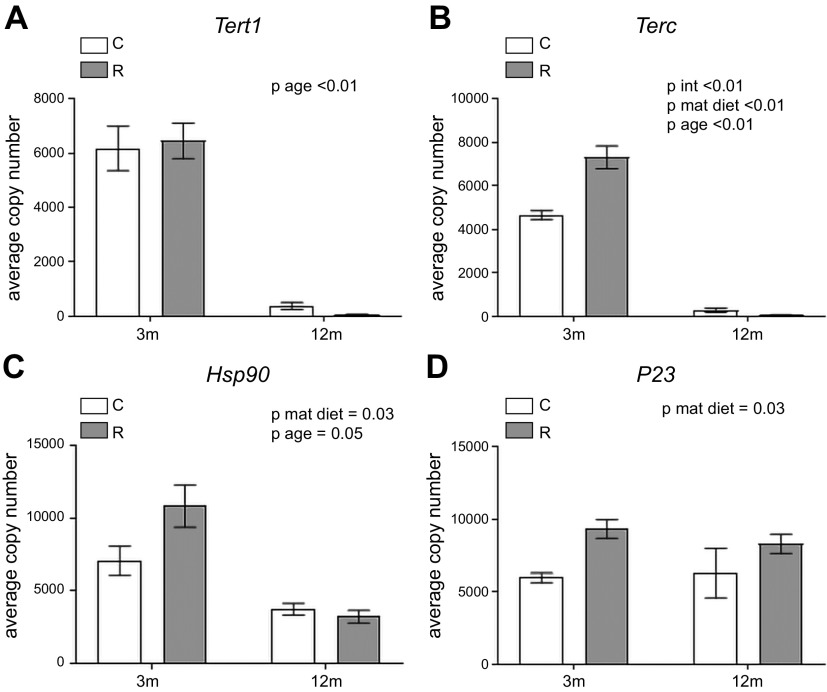
The effect of *in utero* protein restriction, accelerated postnatal growth, and aging upon the expression of telomerase complex molecules in the thymus of 3- and 12-mo-old male rats. *Tert1* (*A*), *Terc* (*B*), *Hsp90* (*C*), and *P23* (*D*). Results are expressed as means ± sem; *n* = 8/group. C, control; R, recuperated. *P* adj-int <0.01; *P* mat diet <0.03 and 0.01; *P* age <0.01 and 0.05.

There was no effect of maternal diet upon telomerase reverse transcriptase-1 (*Tert1*) mRNA expression; however, there was an overall effect of age (*P* < 0.001), with expression of *Tert1* being reduced at 12 mo compared with 3 mo of age (**Fig. 6*A***). There was an overall effect of maternal diet (*P* < 0.001) upon *Terc* expression, with increased expression (*P* < 0.01) in recuperated thymi compared with controls. That increase was only observed at 3 mo, as indicated by the interaction (*P* < 0.01) between maternal diet and age (Fig. 6*B*). Reduced (*P* < 0.01) expression of *Terc* was observed at 12 mo compared with 3 mo of age (Fig. 6*B*). There was an overall effect of maternal diet upon *Hsp90* and *P23* (*P* = 0.03) expression, with increased expression in recuperated thymi compared with controls (Fig. 6*C*, *D*). *Hsp90* was modestly reduced (*P* = 0.05) between 12 and 3 mo of age (Fig. 6C), and there was no effect of age upon *P23* expression (Fig. 6*C*, *D*).

#### Shelterin complex molecules

**Figure 7:**
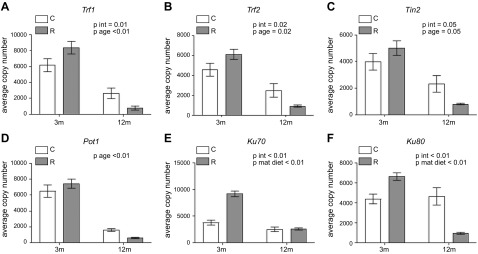
The effect of *in utero* protein restriction, accelerated postnatal growth, and aging upon the expression of shelterin complex proteins in the thymus of 3- and 12-mo-old male rats. *Trf1* (*A*), *Trf2* (*B*), *Tin2* (*C*), *Pot1* (*D*), *Ku70* (*E*), and *Ku80* (*F*). Results are expressed as means ± sem. C, control; R, recuperated; *n =* 8/group. *P* int <0.01, 0.02, and 0.05. *P* mat <0.01; *P* age <0.01.

Interactions between maternal diet and age upon telomere repeat binding factor 1 (*Trf1*; *P* < 0.01), *Trf2* (*P* = 0.02), and Trf1 interacting nuclear factor (*Tin2*; *P* = 0.05) expression highlighted an increased expression at 3 mo, which is reduced by 12 mo in recuperated thymi (**Fig. 7*A–C***). There was no overall effect of maternal diet on protection of telomeres 1 (*Pot1*) expression (Fig. 7*D*). An overall age-associated decrease of *Trf1*; (*P* < 0.01), *Trf2* (*P* = 0.02), *Tin2* (*P* = 0.05), and *Pot1* (*P* < 0.01) was observed (Fig. 7*A–D*). There was also an interaction (*P* < 0.01) between maternal diet and age on the mRNA expression of *Ku70* and *Ku80*, which illustrated an age-associated decrease (*P* < 0.001) in recuperated thymi. That effect was absent in control thymi (Fig. 7*E*, *F*). There was also an overall effect of maternal diet (*P* < 0.001) upon *Ku70* and *Ku80* expression. Recuperated thymi had increased *Ku70* expression at 3 mo old, which disappeared by 12 mo (Fig. 7*E*). There was also increased *Ku80* expression in recuperated thymi at 3 mo old, which, by 12 mo, was decreased compared with controls (Fig. 7*F*).

### Effect of maternal diet and age upon molecular markers of the nonhomologous end joining pathway

**Figure 8:**
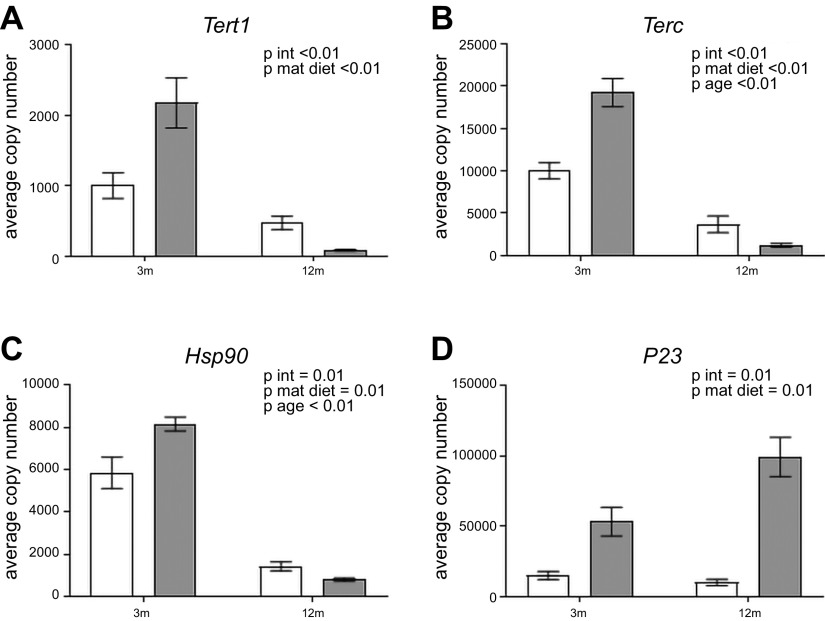
The effect of *in utero* protein restriction, accelerated postnatal growth, and aging upon the expression of markers of DNA damage repair in the thymus of 3- and 12-mo-old male rats. *DNA-PKcs* (*A*), *Mre11* (*B*), *Xrcc4* (*C*), and *γH2ax* (*D*). Results are expressed as means ± sem; *n* = 8/group. C, control; R, recuperated. *P*adj-int < 0.01 and 0.03. *P* mat diet <0.01, 0.01, and 0.02; *P* age <0.01.

An interaction between maternal diet and age upon DNA-dependent protein kinase (*DNA-PKcs*; *P* < 0.01), meiotic recombination 11 (*Mre11*; *P* < 0.01), and X-ray repair complementing defective repair in Chinese hamster cells 4 (*Xrcc4*; *P* = 0.03) was found, with increased expression of those molecules in the recuperated group at 3 mo, which was absent at 12 mo (**Fig. 8*A–C***). There was an overall effect of maternal diet upon *DNA-PKcs* (*P* < 0.01), *Mre11* (*P* < 0.01), and *Xrcc4* (*P* = 0.02) expression (Fig. 8*A–C*). There was also an overall effect of age on *Mre11* and *Xrcc4* expression (*P* < 0.001), with those genes being reduced between 3 and 12 mo of age (Fig. 8*A–C*). Expression of H2A histone family member X (*γH2ax*, a marker of double-stranded DNA breaks) was up-regulated with age in recuperated thymi; however, that effect was absent in control thymi (Fig. 8*D*), as indicated by the interaction of maternal diet and age (*P* = 0.01). *γH2ax* expression was, therefore, increased in recuperated thymi at 3 and 12 mo compared with controls, as indicated by the overall effect (*P* = 0.01) of maternal diet (Fig. 8*D*).

### Significant correlations exist between dsDNA damage marker and telomere shortening

**Figure 9:**
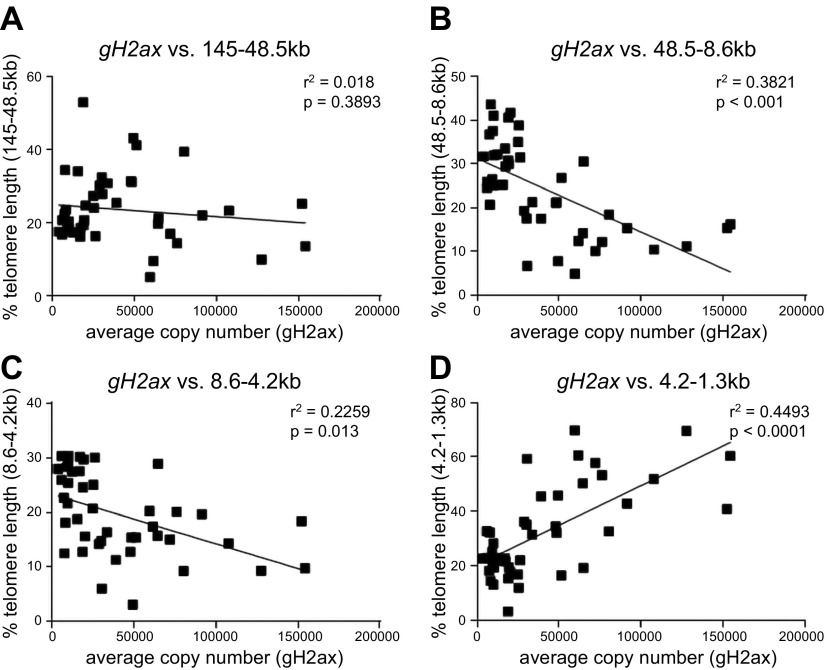
Correlations of thymic telomere length analysis *vs.* gene expression of *γH2ax* in 3- and 12-mo-old male rats. *A*) *γH2ax* *vs.* 145–48.5 kb. *B*) *γH2ax* *vs.* 48.5–8.6 kb. *C*) *γH2ax* *vs.* 8.6–4.2 kb. *D*) *γH2ax* *vs.* 4.2–1.3 kb. Results are expressed as means ± sem; *n =* 8/group. C, control; R, recuperated.

*γH2ax* expression did not correlate with the frequency of the longest (145–8.6 kb) telomere fragments (*r*^2^ = 0.02; *P* = 0.40) (**Fig. 9*A***); however, a negative correlation (*r*^2^ = 0.38; *P* < 0.001) existed between the frequency of long (48.5–8.6 kb) thymic telomeres and *γH2ax* expression (Fig. 9*B*). The intermediate-short telomere (8.6–4.2 kb) fragments were also modestly negatively correlated with *γH2ax* expression (*r*^2^ = 0.23; *P* = 0.01) (Fig. 9*C*). A strong positive correlation was found between *γH2ax* expression and the frequency of the shortest (4.2–1.3 kb) telomeres (*r*^2^ = 0.45; *P* < 0.001) (Fig. 9*D*).

### Effect of maternal diet and age upon sources of nonmitochondrial sources of ROS and antioxidant-defense capacity in rat thymus

**Figure 10:**
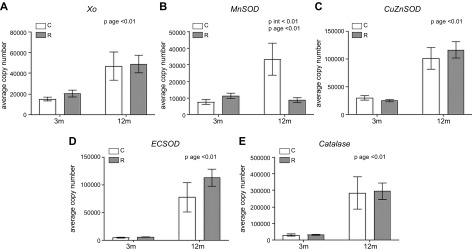
The effect of *in utero* protein restriction, accelerated postnatal growth, and aging upon the expression of sources of nonmitochondrial ROS and antioxidant-defense capacity in the thymus of 3- and 12-mo-old male rats. *Xo* (*A*), *MnSOD* (*B*), *CuZnSOD* (*C*), *ECSOD* (*D*), *Catalase* (*E*). Results are expressed as means ± sem; *n* = 8.group. C, control; R, recuperated. *P* int <0.01; *P* age <0.01.

There was no overall effect of maternal diet upon xanthine oxidase (*Xo*) gene expression; however, there was an overall effect (*P* < 0.001) of age upon *Xo* expression, with *Xo* being increased between 3 and 12 mo of age (**Fig. 10*A***). No effect of maternal diet or age on *Gp91^phox^* (3-mo control: 17,241 ± 2402, 3-mo recuperated: 17,685 ± 646, 12-mo control: 25,240 ± 13,406, and 12-mo recuperated: 18,808 ± 9164) or *P22^phox^* (3-mo control: 19,907 ± 2873, 3-mo recuperated: 19,978 ± 3892, 12-mo control: 17,931 ± 6911, and 12-mo recuperated: 9507 ± 2708 copy numbers) was observed. There was an interaction (*P* < 0.01) between maternal diet and age upon manganese superoxide dismutase (*MnSOD*) expression, which reflected an age-associated increase of *MnSOD* in control thymi that was absent in recuperated thymi (Fig. 10*B*). Consequently, *MnSOD* was unchanged in recuperated thymi at 3 mo of age; however, by 12 mo, *MnSOD* expression was robustly reduced in recuperated thymi compared with controls (Fig. 10*B*). There was no effect of maternal diet upon *CuZnSOD*, *ECSOD*, or *Catalase* expression (Fig. 10*C–E*); however, there was an overall effect of age (*P* < 0.01), with an observed age-associated increase in those molecules (Fig. 10*C–E*).

### Effect of maternal diet and age upon mitochondrial function in rat thymus

There was no overall effect of maternal diet upon expression of complex I *(Ndufa5*), complex II (*Sdha*), complex III (*Uqcrc1*), and complex IV (*Cox4i1*) of the mitochondrial electron transport chain (**Table 4**). There was an interaction between maternal diet and age upon *Uqcrc1* and uncoupling protein-1 (*Ucp1*; *P* < 0.01) expression. That highlighted increased *Uqcrc1* and *Ucp1* in recuperated thymi compared with controls occurred at 12 mo only (Table 4). There was an overall effect of age on *Ndufa5* (*P* < 0.01), *Sdha* (*P* < 0.01), *Uqcrc1* (*P* = 0.02), *Cox4i1* (*P* < 0.01), and citrate synthase (*Cs*; *P* < 0.01) expression, with those molecules being increased between 3 and 12 mo of age (Table 4). Maternal diet had no effect on *Cs* expression (Table 4).

**TABLE 4:** The effect of maternal diet and accelerated postnatal growth upon markers of mitochondrial ROS and antioxidant-defense capacity in thymi from male rats

Gene	Control 3 mo	Recuperated 3 mo	Control 12 mo	Recuperated 12 mo
*Ndufa5*	21,722 ± 3110	19,107 ± 1151	130,298 ± 38,227***	125,259 ± 9614***
*Sdha*	18,365 ± 3855	18,238 ± 1624	150,711 ± 54,513***	88,895 ± 13,166***
*Uqcrc1*	34,909 ± 7696	31,257 ± 2838	112,216 ± 33,447***	243,212 ± 29,488***
*Cox4i1*	50,206 ± 9396	46,685 ± 4940	246,057 ± 77,736***	394,701 ± 31,326***
*Cycs*	24,647 ± 4633	33,474 ± 8457	40,174 ± 3819***	67,169 ± 8942***
*Ucp1*	1851 ± 921	2465 ± 792	36,498 ± 13,385***	131,345 ± 49,643***
*Cs*	21,162 ± 4062	20,529 ± 1961	155,159 ± 48,812***	136,928 ± 16,725***

Overall effect of age: ***P < 0.001 3- *vs.* 12 mo old. Interaction between maternal diet and age: *Cyc1* and *Ucp1* (*P* < 0.01). Cycs, cytochrome *c.*

## DISCUSSION

A suboptimal maternal diet during pregnancy, followed by a mismatched postnatal milieu increases, the risk of developing age-associated diseases; however, the extent that developmental programming influences immunodeficiency and immunosenescence is unclear. Therefore, we investigated whether/how alterations in the early life environment can contribute to changes in age-associated changes in immune function with a rat model of maternal protein restriction and accelerated postnatal growth.

Analysis of thymus ultrastructure by histologic assessment revealed the expected age-associated reduction in total thymus, cortex, and medulla areas (27, 28). That was associated with a robust increase in adipose tissue area and adipocyte number, which likely contributes to the increased thymic mass with age. Recuperated thymi also demonstrated increased adipose tissue area and had larger adipocytes in the absence of adipocyte hyperplasia. Increased *Lep* mRNA expression was also found in recuperated thymi. Our data reflect previous studies in which leptin associated positively with adipocyte size (29). There was also an overall age-associated increase in adipocyte area, which was more apparent in control thymi. This may suggest that adipocyte size had reached its threshold limit in recuperated thymi in early life (3 mo) and, therefore, could not grow any further. Conversely, the control thymi may have been undergoing a normal age-associated increase in adipocyte area. Taken together, these data suggest that developmentally programmed thymi demonstrate increased adipocyte infiltration, a well-recognized marker of thymi involution.

Maternal diet did not significantly alter cortical area; however, the age-associated decline in the medullary area was less apparent in recuperated thymi. An important hallmark of thymic involution is the alteration of the C/M (27, 28). We demonstrated an overall decline in C/M with age, which is consistent with previous reports (27, 28). That decrease was more apparent in recuperated thymi, consistent with accelerated aging. Consequently, thymi from 12-mo-old, recuperated offspring demonstrated a modest reduction in the C/M compared with control thymi. To further support that finding, we calculated C/T as a proxy for cortical thinning. An age-associated decline in that ratio was only observed in recuperated offspring, resulting in a reduction in that ratio in the elderly, recuperated thymi, again consistent with accelerated age-associated thymic involution in recuperated animals. Taken together, we have demonstrated that a poor maternal diet and accelerated postnatal growth influences age-associated changes in thymic ultrastructure, which affect thymic involution.

We also demonstrated a robust age-associated decline in the expression of markers of TEC lineage (*Tcf3*, *Krt8*, *Il7*, and *Foxn1*), which have an important role in thymic involution and are essential for maintenance of thymic architecture, development, function, maintenance, and regeneration (13, 14). We also observed increased *Tcf3*, *Il7*, and *Foxn1* in 3-mo-old recuperated thymi. That may indicate an initial compensatory preservation of thymic integrity in young recuperated rats (which may explain why we observed no significant changes to thymic ultrastructure at that age). The age-associated decline in TEC markers was much more apparent in recuperated thymi, consistent with accelerated thymic involution. We also demonstrated an overall age-associated decline in markers of thymocyte lineage (*Cd8*, *Cd44*, and *Cd69*). That observation is consistent with previous findings by Aspinall (30) in mice that showed thymocyte number decreased 83% between 3 and 20 mo of age. Recuperated thymi also demonstrated overall reduced expression of those thymocyte lineage markers, which may suggest accelerated aging of recuperated thymi *via* a reduction in naive T-cell output. We acknowledge, however, that the lack of immunofluorescent assessment of thymic ultrastructure using keratin 5 and 8 as markers may be a limitation to this study. Unfortunately, that assessment was not feasible because of technical issues involving the unsuitability of several antibodies.

Telomere length is a known marker of cellular age (17). The thymus is a complex tissue with which to explore telomere length because telomerase (a positive regulator of telomere length) is expressed in young thymic tissue (31). Control thymic telomeres lengthened between 22 d and 3 mo but then shortened between 3 and 12 mo. Thymic telomere elongation in early life has been previously reported by Weng *et al.* (31) and occurs so thymocytes can acquire telomere sequences that are long enough to undergo several rounds of replication, and that process is positively associated with T-cell lineage development and activation. In old thymi, T-cell proliferation and differentiation declines (31), consistent with our observation that expression of the telomerase components *Tert1* and *Terc* were down-regulated in control thymi at 12 mo compared with 3 mo. The pattern of age-associated telomere-length alteration differed in recuperated thymi: telomeres in that group were already longer than controls at 22 d and then shortened consistently during the aging process, which was associated with an age-associated decrease in *Tert1* and *Terc* expression. The different rates of age-associated telomere attrition meant that 3-mo-old recuperated rats maintained longer thymic telomeres compared with age-matched control littermates. That was supported by increases in *Terc* expression in recuperated thymi at 3 mo, which can act independent of *Tert1* (which remained unchanged) (32). *P23* and *Hsp90*, which are also required for the functional assembly of telomerase (33), were up-regulated in recuperated thymi at 3 mo of age. That may also be directly related to the *Terc* increase in young recuperated animals. By 12 mo, recuperated thymic telomeres were shorter than those of controls, which suggests an accelerated aging phenotype in the recuperated group.

A major mechanism in telomere length regulation and stability is the shelterin complex. This complex coordinates the formation of the protective T-loop structure in telomeres and is made up of subunits, including *Trf1*, *Trf2*, *Tin2*, and *Pot1*. When telomeres elongate, *Trf1* encodes part of the nucleoprotein complex that binds to telomeric repeats to prevent their degradation (34). *Trf2* is also essential for telomere protection and chromosomal stability (35), and both *Trf1* and *Trf2* bind to *Tin2* to facilitate telomere protection. At 3 mo of age, *Trf1*, *Trf2*, and *Tin2* expression was increased in recuperated thymi but was reduced compared with controls at 12 mo, which is in keeping with the preservation of thymic telomere length in young recuperated thymi that then undergo accelerated shortening in old age. We also found an overall age-associated reduction in *Trf1*, *Trf2*, *Tin2*, and *Pot1* expression, which was in keeping with the observed age-associated telomere shortening. Other molecules that have an integral role in the preservation of telomere integrity are *Ku70* and *Ku80*, which can interact with *Trf1* to prevent end joining of telomeres (36); 3-mo-old recuperated thymi had increased expression of *Ku70* and *Ku80*, which, again, may be indicative of an early life compensatory response. However, by 12 mo, that increase had disappeared, and in the case of *Ku80*, it was robustly decreased, which may indicate telomere damage and shortening in old recuperated thymi. Taken together these data suggest that young recuperated thymic telomeres are protected, in part, by regulation of the shelterin complex; however, by 12 mo, that protection disappeared and, consequently, accelerated telomere shortening was observed.

The shelterin complex also prevents dsDNA-break damage sensing machinery, such as the nonhomologous end-joining pathway (NHEJ), from mistakenly repairing telomeres. We observed increased expression of the NHEJ molecules *DNA-PKcs*, *Mre11*, and *Xrcc4* in recuperated thymi at 3 mo of age, which decreased by 12 mo. At first glance, that may seem counterintuitive within the context of increased expression of some molecules of the shelterin complex. However, that increase was accompanied by increased expression of *γH2ax*, a marker of dsDNA damage. Therefore, it is plausible that increased NHEJ pathway components are targeting the *γH2ax-*mediated DNA damage. *γH2ax* foci have a robust correlation with replicative telomere shortening (16), as demonstrated by a positive correlation between *γH2ax* expression and thymic telomere shortening in this study. *DNA-PKcs*, *Mre11*, and *Xrcc4* were also markedly reduced at 12 mo, which is consistent with previous findings by Gorbunova *et al.* (37) showing that the NHEJ DNA repair mechanism declines with old age, and that decline has been associated with increased genomic instability (37, 38).

ROSs are major contributors to telomere shortening and accelerated aging (20, 39). An increase in *Xo*, a source of ROS, was observed between 3 and 12 mo of age. In 3-mo-old recuperated thymi, expression of the mitochondrial antioxidant defense enzyme *MnSOD* was unchanged compared with controls. However, the age-related increase in *MnSOD* expression observed in control thymi was not observed in the recuperated group, suggesting that recuperated thymi were unable to mount an appropriate mitochondrial antioxidant defense response to the age-associated increase in *Xo*. That dysfunction was specific to the mitochondria because *CuZnSOD*, *ECSOD*, and *Catalase* expression were unchanged. *Ucp1*, the mitochondrial uncoupling protein, is classically expressed in brown adipose tissue; however, *Ucp1* has also been found in rodent thymocytes (40, 41). In the current study, *Ucp1* expression was increased in 12-mo-old recuperated thymi. Given that a murine thymic *Ucp1* knockout model demonstrated decreased T-cell apoptosis (42), that increase may confer a potential for increased T-cell apoptosis in old recuperated rats.

A potential limitation of our study is that we did not carry out immunofluorescent assessment of thymic ultrastructure and only performed hematoxylin and eosin staining (to assess thymic ultrastructure and integrity) and Sudan Black staining (to assess cellular senescence). Immunostaining using antibodies to keratin 5 and 8 could have provided more information on changes in thymic architecture. However, that was not possible following the protocol we used for tissue collection and processing.

In summary, a suboptimal early life environment induced initial mechanisms to protect thymic integrity, which included elongation of thymic telomere length and increased DNA damage repair. Those changes may relate to preservation in thymic ultrastructure at that age. By 12 mo, an accelerated aging phenotype was observed in recuperated thymi, which included accumulation of adipose tissue, alterations in thymic ultrastructure, decreased markers of TECs and thymocytes, accelerated telomere shortening, alterations in antioxidant defense, increased DNA damage, and reduced expression of molecules involved in DNA-damage repair. Those changes seem to be independent of alterations in cellular senescence; however, we cannot discount the possibility of them occurring in older rats. The thymus is unique because it is largest early in life and undergoes a rapid functional and structural decline with age. Interestingly, in the face of this strong phenotype, we have shown that alterations in the early life milieu affect the markers of thymic integrity. Those data, therefore, highlight the necessity for longer-term immunologic studies of “programmed” individuals because it is clear that aging has a critical role in mediating the detrimental effects of thymic involution in rats from a suboptimal early life environment.

## Supplementary Material

This article includes supplemental data. Please visit *http://www.fasebj.org* to obtain this information.

## Abbreviations

C/M: thymic cortex to medulla area
*Cs*: citrate synthase
C/T: cortex to total thymus area
*DNA-PKcs*: DNA-dependent protein kinase, catalytic subunit
dsDNA: double-stranded DNA
*Foxn1*: forkhead box N1
*Glut4*: glucose transporter 4
*γH2ax*: H2A histone family member X
*Krt8*: keratin 8
*Lep*: leptin
*MnSOD*: manganese superoxide dismutase
*Mre11*: meiotic recombination 11
NHEJ: nonhomologous end joining
*Pot1*: protection of telomeres 1
ROS: reactive oxygen species
*Tcf3*: transcription factor 3
TEC: thymic epithelial cell
*Terc*: telomerase RNA component
*Tert1*: telomerase reverse transcriptase-1
*Tin2*: Trf1 interacting nuclear factor
*Trf*: telomere repeat binding factor
*Ucp1*: uncoupling protein-1
*Xo*: xanthine oxidase
*Xrcc4*: X-ray repair complementing defective repair in Chinese hamster cells 4
