# Supplementary material for: A suboptimal maternal diet combined with accelerated postnatal growth results in an altered aging profile in the thymus of male rats
Source: FASEB J. 2018 Jul 5;33(1):239–53. doi: 10.1096/fj.201701350RR (PMC6314471; doi:10.1096/fj.201701350RR)
Supplement: Supplementary file 1 [file fj.201701350RR.sf1.pptx]

## Slide 1
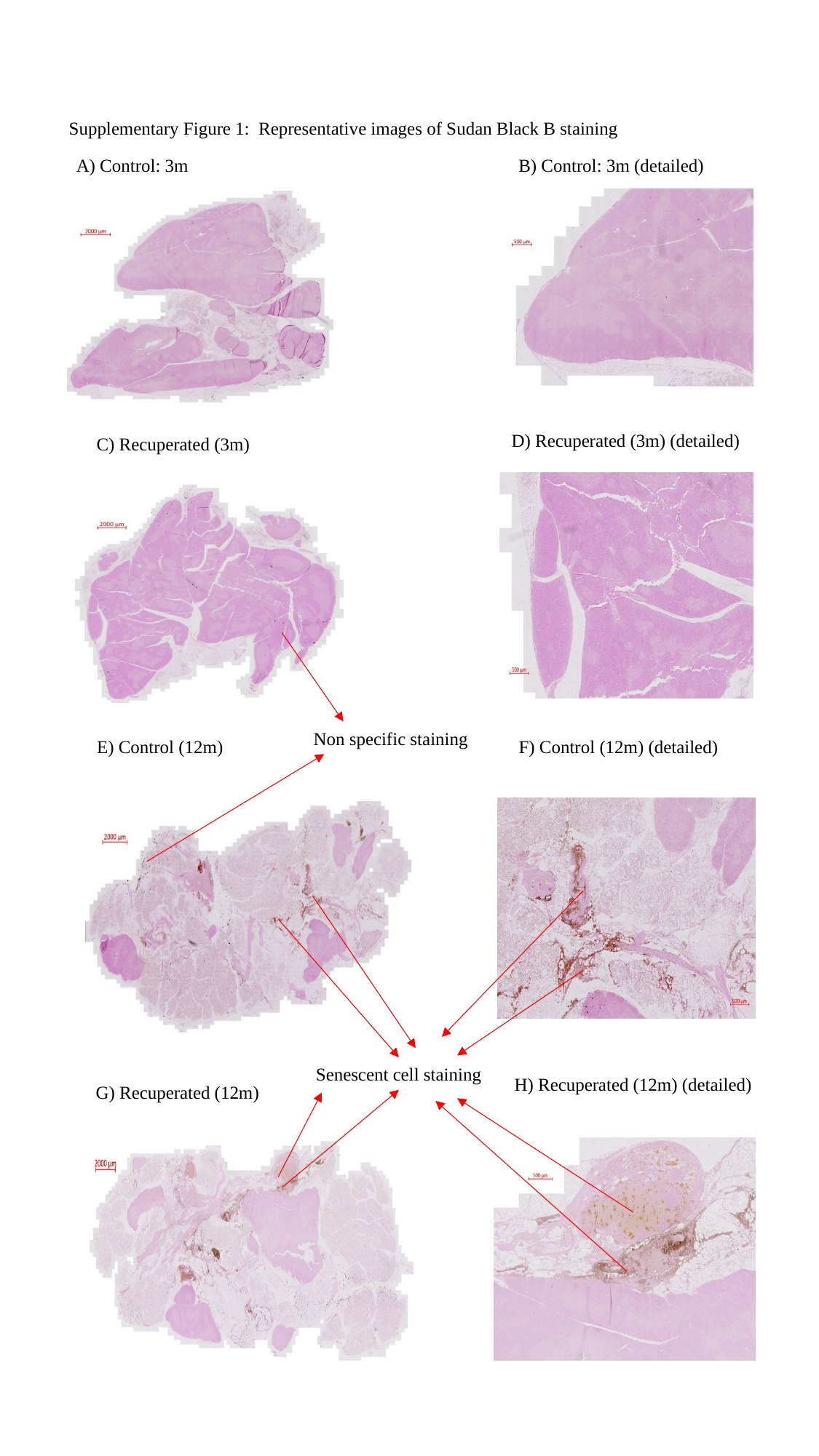

Supplementary Figure 1: Representative images of Sudan Black B staining
A) Control: 3m
B) Control: 3m (detailed)
D) Recuperated (3m) (detailed)
C) Recuperated (3m)
Non specific staining
E) Control (12m)
F) Control (12m) (detailed)
Senescent cell staining
H) Recuperated (12m) (detailed)
G) Recuperated (12m)
