# Supplementary material for: A suboptimal maternal diet combined with accelerated postnatal growth results in an altered aging profile in the thymus of male rats
Source: FASEB J. 2018 Jul 5;33(1):239–53. doi: 10.1096/fj.201701350RR (PMC6314471; doi:10.1096/fj.201701350RR)
Supplement: Supplementary file 2 [file fj.201701350RR.st1.docx]

Supplementary Table 1: Primer sequences

| ***Gene*** | **Forward Sequence** | **Reverse Sequence** | **Product size (kb)** |
| --- | --- | --- | --- |
| ***Leptin*** | AAGCCTCGCTCTACTCCACA | TCAGCATTCAGGGCTAAGGT | 85 |
| ***Glut4*** | CCTGCTTGGCTTCTTCATCT | AGAAGGTGTCCGTCGGAAG | 97 |
| ***Tert1*** | CTGGCAGGTATACGGCTTTC | CTTTAAGAAGCGGCGCTCA | 92 |
| ***Terc*** | CCGCCGTGAAGAGCTAGT | TCAGGGACCAGTTCCGTTAC | 97 |
| ***Trf1*** | TCAACAACCGAACAAGTGTCA | TGAGCCTTAGCCTCCTCATC | 63 |
| ***Trf2*** | CATCGGACTCAGGACAGTGA | TCTTTCTGGTCCAGTTTTGC | 90 |
| ***Tin2*** | TAGCTGGCAACCGCTTTAAT | CTCTTTGTGGCAGGCCTTT | 86 |
| ***Hsp90*** | AAGGCAGAGGCTGACAAGAA | AGACTGAAGCCGGAAGACAG | 89 |
| ***P23*** | GGCCTCCTCCTTTAGTGCTT | AGCTGCTCAAATGCCTCATC | 90 |
| ***Pot1*** | CTGCAACTAAAGCGCCAGAC | CGGTGGTCCAGATCTTTGAT | 69 |
| ***Ku70*** | ACTGAGGGACATCTGCAAGG | TCCAAGTGTCTGCTGAGAGC | 84 |
| ***Ku80*** | GACATGAAGCTCTGGCCATC | TGTCTGTAGGGACCTGGAGTG | 65 |
| ***DNA PKcs*** | CAACATTCGTGCACAAGAGC | CTGATCCACCAAGCACTTCA | 70 |
| ***Mre 11*** | CTCAGCCTTCAGTGCAGATG | CACTGCTGACTGGCTATCGT | 82 |
| ***Xrcc4*** | TTGCACCAAGTAGGAAGAGGA | TCCTGTGGAGCCATTTTAGG | 73 |
| ***γH2AX*** | AACGACGAGGAGCTCAACAA | CTGGATGTTGGGCAGGAC | 72 |
| ***Tcf3*** | GAGAAGGTATCTGGCGTGGT | GTGCCCAGCTGGATTGTG | 90 |
| ***Lmo2*** | TGCCTATGAGATGACGATGC | GGCAGCGCACTTGAAAC | 68 |
| ***Krt8*** | CCGGCTTCAGCTATGGAAT | CCTTGGTGCGGCTATAAGTG | 81 |
| ***Il7*** | GTGCTGCTCGCAAGTTGA | TTGTGTGCCGTCTGAAACTC | 89 |
| ***Foxn1*** | GCTAGGATGTCCACCACCTG | GGATGCATTGGGTGTAGAGG | 96 |
| ***Tmpo*** | CCGTTCCCATGTGGATAAAA | CCTTGGTTGGTTTCCATAGC | 88 |
| ***Xo*** | GAGAAGGTCTCCAGCAGTGG | GCATGCGGAAATCTGGATA | 86 |
| ***Gp91phox*** | CGAAGCCTTGGCTAAAACTCT | TCCTTGTTGAAGATGAAGTGGA | 87 |
| ***P22phox*** | GTGAGCAGTGGACTCCCATT | GTAGGTGGCTGCTTGATGGT | 76 |
| ***MnSOD*** | TGACTATGTAATGTTTTATCAGTTGGA | GTTGCTGACCACAGCCTTTT | 91 |
| ***CuZnSOD*** | TTGTGGTGTGATTGGGATTG | CAGTTTAGCAGGACAGCAGATG | 80 |
| ***ECSOD*** | ATCCCATAAGCCCCTAGCAT | ATTCGACCTCTGGGGGTAAG | 84 |
| ***Catalase*** | TTGGATCATGTCTTCCAAAAA | GGGAAAAGGAATCCGATCAA | 83 |
| ***P53*** | CCTATCCGGTCAGTTGTTGG | CGTATGAGGGCCCAAGATAG | 89 |
| ***P21*** | TGCAAGAGAAAGCCCTGAAG | TGAATGAAGGCTAAGGCAGAA | 96 |
| ***Ndufa5*** | TGGCAAGAAAAATGTTGCAG | TATTGGCCACTTCCACTGGT | 80 |
| ***Sdha*** | GGATCAGATTGTGCCTGGTC | TCCAAAAGAGAGTTTGCTCCA | 93 |
| ***Uqcrc1*** | AAGCTTTGCCAGAGTTTCCA | ATGCTCATGGCATCACAGAC | 89 |
| ***Cox4i1*** | TCCCTCATACCTTTGATCGTG | GTTGACCTTCATGTCCAGCA | 68 |
| ***Cycs*** | GGGAGAGGATACCCTGATGG | TGCCCTTTCTCCCTTCTTCT | 100 |
| ***Cs*** | TCCTGTTCGGAGTGTCGAG | CATGGACTTGGGCCTTTCTA | 92 |
| ***Ucp1*** | CCGGTGGATGTGGTAAAAAC | TTGCACAGCTGGGTACACTT | 73 |
| ***Cox1*** | AGACACCTCTCTTTGTATGATCC | CGGTCTGTAAGGAGTATAGTGAT | 89 |
| ***Nthl*** | GATTTGCCTTCCTGTCCATC | GAAGCCCAAAACCCTCAGA | 90 |
| ***Cd8*** | CAGAAAGGCTTGACATGTGG | CACTGAGGGATACCAGCAGAA | 79 |
| ***Cd69*** | TTTAACAGCTGGTTCAACGT | TGTAAGTTTGCCTCGCAGTC | 92 |
| ***Cd44*** | CTCAACGGGGAGGCCAGC | CCGGAGTCTCTGTTGGTTCC | 70 |
| ***Ppia*** | TGAGAACTTCATCCTGAAGCATACA | CATTTGTGTTTGGTCCAGCATT | 89 |
