# Supplementary material for: A suboptimal maternal diet combined with accelerated postnatal growth results in an altered aging profile in the thymus of male rats
Source: FASEB J. 2018 Jul 5;33(1):239–53. doi: 10.1096/fj.201701350RR (PMC6314471; doi:10.1096/fj.201701350RR)
Supplement: Supplementary file 3 [file fj.201701350RR.st2.docx]

Supplementary Table 2

Alpha levels derived from linear regression modelling for mRNA data, following adjustment for multiple hypothesis testing.

| Gene name | Maternal Diet | | Age | | | Interaction term between maternal diet and age |
| --- | --- | --- | --- | --- | --- | --- |
|  | Control | Recuperated | 22 days | 3 months | 12 months |  |
| *Lep* | Ref. | *0.06* | Ref. | 0.99 | <0.01 | 0.22 |
| *Glut4* | Ref. | 0.99 | Ref. | 0.99 | 0.18 | 0.23 |
| *Tert1* | Ref. | 0.99 | Ref. | 0.99 | <0.01 | 0.62 |
| *Terc* | Ref. | <0.01 | Ref. | 0.99 | <0.01 | <0.01 |
| *Hsp90* | Ref. | 0.03 | Ref. | 0.99 | *0.05* | *0.08* |
| *P23* | Ref. | 0.03 | Ref. | 0.99 | 0.84 | 0.59 |
| *Trf1* | Ref. | *0.08* | Ref. | 0.99 | <0.01 | 0.01 |
| *Trf2* | Ref. | 0.13 | Ref. | 0.99 | 0.02 | 0.02 |
| *Tin2* | Ref. | 0.35 | Ref. | 0.99 | *0.05* | *0.05* |
| *Pot1* | Ref. | 0.99 | Ref. | 0.99 | <0.01 | *0.08* |
| *Ku70* | Ref. | <0.01 | Ref. | 0.99 | *0.07* | <0.01 |
| *Ku80* | Ref. | <0.01 | Ref. | 0.99 | 0.75 | <0.01 |
| *DNA Pkcs* | Ref. | <0.01 | Ref. | 0.99 | 0.19 | <0.01 |
| *Mre11* | Ref. | <0.01 | Ref. | 0.99 | <0.01 | <0.01 |
| *Xrcc4* | Ref. | 0.02 | Ref. | 0.99 | <0.01 | 0.03 |
| *γH2ax* | Ref. | 0.01 | Ref. | 0.99 | 0.74 | 0.01 |
| *Tcf3* | Ref. | 0.41 | Ref. | 0.99 | 0.54 | *0.05* |
| *Krt8* | Ref. | 0.15 | Ref. | 0.99 | <0.01 | 0.14 |
| *Il7* | Ref. | 0.01 | Ref. | 0.99 | <0.01 | 0.02 |
| *Foxn1* | Ref. | <0.01 | Ref. | 0.99 | <0.01 | <0.01 |
| *Xo* | Ref. | 0.92 | Ref. | 0.99 | <0.01 | 0.82 |
| *Gp91^phox^* | Ref. | 0.99 | Ref. | 0.99 | 0.5 | 0.67 |
| *P22^phox^* | Ref. | 0.99 | Ref. | 0.99 | 0.75 | 0.37 |
| *MnSOD* | Ref. | 0.92 | Ref. | 0.99 | <0.01 | <0.01 |
| *CuZnSOD* | Ref. | 0.99 | Ref. | 0.99 | <0.01 | 0.46 |
| *ECSOD* | Ref. | 0.99 | Ref. | 0.99 | <0.01 | 0.27 |
| *Catalase* | Ref. | 0.99 | Ref. | 0.99 | <0.01 | 0.94 |
| *Ndufa5* | Ref. | 0.32 | Ref. | 0.99 | <0.01 | 0.94 |
| *Sdha* | Ref. | 0.99 | Ref. | 0.99 | <0.01 | 0.25 |
| *Uqcrc1* | Ref. | 0.99 | Ref. | 0.99 | 0.02 | <0.01 |
| *Cox4i1* | Ref. | 0.99 | Ref. | 0.99 | <0.01 | *0.07* |
| *Ucp1* | Ref. | 0.99 | Ref. | 0.99 | 0.62 | <0.01 |
| *Cycs* | Ref. | 0.70 | Ref. | 0.99 | 0.19 | 0.30 |
| *Cs* | Ref. | 0.99 | Ref. | 0.99 | <0.01 | 0.75 |
